# The Presence of Bioactive Compounds in European Eel (*Anguilla anguilla*) Skin: A Comparative Study with Edible Tissue

**DOI:** 10.3390/md22030105

**Published:** 2024-02-24

**Authors:** Antía Bote, Marcos Trigo, Sidonia Martínez, Santiago P. Aubourg

**Affiliations:** 1 Marine Research Institute (CSIC), c/E. Cabello, 6, 36208 Vigo, Spain; antia.bote.chamorro@alumnos.uvigo.es (A.B.); mtrigo@iim.csic.es (M.T.); 2 Food Technology, Faculty of Science, University of Vigo, 32004 Ourense, Spain; sidonia@uvigo.gal

**Keywords:** European eel (*Anguilla anguilla*), skin, muscle, proteins, phospholipids, free sterols, α-tocopherol, ω3 fatty acids, ω3/ω6 ratio, source

## Abstract

The presence of bioactive compounds in European eel (*Anguilla anguilla*) skin was studied. Proximate and lipid class compositions and analysis of the fatty acid (FA) profile (individual FAs; FA groups, i.e., saturated, monounsaturated, and polyunsaturated; FA ratios, i.e., polyunsaturated/saturated, ω3/ω6) were determined and compared to the composition of the eel muscle. As a result, higher (*p* < 0.05) levels of proteins (271.6 g·kg^−1^), lipids (38.0 g·kg^−1^), ash (27.7 g·kg^−1^), and ω6 FAs were observed in the skin tissue. Contrary, the muscle tissue showed higher (*p* < 0.05) moisture, ω3 FA, and ω3/ω6 ratio values. Regarding lipid classes, a higher (*p* < 0.05) proportion of phospholipids (111.1 g·kg^−1^ lipids), free sterols (104.7 g·kg^−1^ lipids), α-tocopherol (274.0 mg·kg^−1^ lipids), and free FAs (43.6 g·kg^−1^ lipids) was observed in the skin tissue. No differences (*p* > 0.05) between both tissues could be detected for triacylglycerol and FA group (saturated, monounsaturated, and polyunsaturated) values and for the polyunsaturated/saturated FA ratio. It is concluded that European eel skin, a by-product resulting from commercial processing, can be considered a valuable source for the food and pharmaceutical industries by providing value-added constituents such as proteins, lipids, ω3 FAs, phospholipids, and α-tocopherol.

## 1. Introduction

A wide range of studies have recognised the fish fatty acid (FA) profile and the lipid class composition as being responsible for the health benefits resulting from the employment of fish-enriched diets [1,2]. Among polyunsaturated FAs (PUFAs), eicosapentaenoic (EPA) and docosahexaenoic (DHA) acid consumption has been associated with a low prevalence of several human diseases such as cardiovascular and neurodegenerative concerns [3,4]. According to their amphiphilic character, phospholipid (PL) compounds have shown to be valuable drug delivery systems for their high bioavailability and protecting effects on different kinds of diseases [5,6]. Regarding tocopherol compounds, fishery products have been described as an important source of this effective lipid-soluble antioxidant system [7,8].

As a result of fish processing, a large volume of undesired by-products is obtained that constitute an important environmental contamination source, unless efforts for their recovery are attained and their commercial value can be enhanced [9,10]. Remarkably, fish by-products have been reported to include bioactive and profitable components such as amino acids, enzymes, collagen, pigments, chitin, vitamins, and minerals [11,12]. Among fish by-products, skin tissue has attracted a great attention and has been studied in different kinds of fish species. Thus, Spanish mackerel (*Scomberomorous niphonius*) [13] and great hammerhead shark (*Sphyrna lewini*) [14] skin showed to be a remarkable source of collagen. Additionally, skin tissue from bigeye snapper (*Priacanthus tayenus* and *Priacanthus macracanthus*) [15], snakehead, and shark [16] showed to be a valuable substrate for gelatine extraction. Notably, fish skin-derived peptides have shown antioxidant [17,18,19], antimicrobial [20], and antifreezing [21] properties.

European eel (*Anguilla anguilla*), a teleost fish belonging to the family *Anguillidae*, is a commercially valuable species in Europe and Asia. As a result of recent overfishing on coasts and several biological concerns [22], great attention has been accorded to the development of this fish species as a farmed product [23,24]. With the extension of farming, the total annual production is assumed to be over 10,500 tonnes, The Netherlands consuming ca. 50% of this [25]. Previous studies account for the analysis of the proximate composition and FA profile [26,27] and the presence of essential and toxic elements in the muscle [28]. Additionally, the evolution of the eel muscle quality has been studied during different processing conditions such as refrigeration [29,30], cooking [31], and canning [32].

However, previous research on European eel by-products can be considered very scarce. Thus, Sila et al. [33] carried out the extraction and characterisation of sulphated glycosaminoglycans. Taktak et al. [34] developed novel eco-friendly, gelatine-based microfibers from eel skin for fish encapsulation. Teng et al. [35] prepared peptide-chelated calcium from European eel bones. Regarding eel skin, it is considered a thick substrate that is commonly treated as a waste material during the commercial processing of eel and is normally converted into low-value products or discarded. The unemployment of this by-product not only results in the loss of a large amount of bioactive constituents, but also leads to environmental concerns.

The current study focused on the presence of bioactive compounds in European eel (*A. anguilla*) skin. Determination of proximate and lipid class compositions and analysis of the FA profile, i.e., individual FAs, FA groups (saturated, STFAs; monounsaturated, MUFAs; PUFAs), and FA ratios (PUFAs/STFAs and ω3 FAs/ω6 FAs) was carried out. On the basis of the wide consumer acceptance of eel muscle as a valuable food for the human diet, the composition of the edible tissue was also analysed in this study and was compared to the composition of the skin.

## 2. Results and Discussion

### 2.1. Determination of the Proximate Composition

Values obtained for the proximate composition are included in Table 1. Water was shown to be the most abundant constituent in both eel tissues, with a higher (*p* < 0.05) value being detected in the muscle. Crude protein levels higher than 160 g·kg^−1^ wet tissue were observed in both tissues; notably, values obtained in the skin tissue (ca. 272 g·kg^−1^ wet tissue) were higher (*p* < 0.05) than in the muscle. The crude lipid content of the present eel samples depicted values included in the 28–38 g·kg^−1^ wet tissue range. As for crude protein content, crude lipid values were found to be higher (*p* < 0.05) in the skin tissue. Regarding the ash content, skin samples (27.7 g·kg^−1^ wet tissue) showed higher values (*p* < 0.05) than their counterparts, corresponding to the muscle tissue (9.9 g·kg^−1^ wet tissue).

The crude protein content obtained in the present study is higher than the one found in the muscle of most commercial fish species [36,37,38]. Therefore, this by-product can be considered a protein-rich substrate. Regarding the current crude lipid content of European eel skin, this substrate maybe ranked as a medium-fat substrate [36] and could be considered a valuable source of lipid components.

To the best of our knowledge, no previous research has focused on the proximate composition of European eel (*A. anguilla*) skin. However, previous research provides information regarding the muscle tissue of this fish species. Thus, higher lipid contents (5.0%) than in the present study were obtained by Özoğul et al. [26] in individuals caught in the northeastern Mediterranean. Additionally, higher protein (19.2–19.6%), lipid (5.0–10.21%), and ash (1.23–1.50%) levels were detected in European eel (*A. anguilla*) muscle when studying freshwater individuals corresponding to several sizes [27].

Previous studies have also addressed the proximate composition of the edible tissue of other eel species. Thus, Oku et al. [39] carried out a comparative study on wild and cultured Japanese eel (*Anguilla japonica*) muscle; as a result, higher protein (19.0 and 18.9%, respectively) and lipid (11.6 and 13.1%, respectively) values than in the present study were obtained, although moisture values were lower (69.1 and 67.4%, respectively). A higher protein content (ca. 18.1%) than in the current study was also detected in farmed and freshwater eel (*Monopterus albus*) muscle [40]. A varying lipid content (3.6–20.4%) resulting from the catching season and location was proved for freshwater eel (*A. japonica*) muscle [41], as well as from comparing *A. japonica* individuals in the initial and terminal stages of spawning migration (0.3–20.6%) [42].

Previous research accounts for the proximate composition of skin in different kinds of fish species. Thus, Njinkoué et al. [43] studied the lipid content of different fish species from the Senegalese coast; according to the present results, higher values were detected in the skin than in the white muscle for *Sardinella maderensis* (26 vs. 5.0%), *Sardinella aurita* (24 vs. 3.5%), and *Cepahlopholis taeniops* (2.4 vs. 1.3%). According to the present results, Pateiro et al. [44] found higher protein (ca. 25%) and lipid (ca. 27%) values in gilthead seabream (*Sparus aurata*) skin than in the counterpart muscle (ca. 21 and 8%, respectively). A similar protein content (ca. 28%) was detected by Ahmmed et al. [45] in blue mackerel (*Scomber australasicus*) skin; however, lower lipid (ca. 21%) and higher moisture (ca. 50%) values were obtained. The same authors (Ahmmed et al. [46]) obtained similar moisture values (ca. 65%) than in the present study in king salmon (*Oncorhynchus tshawytscha*) skin; however, protein and lipid contents were notably lower (20 and 13%, respectively). Recently, Park et al. [47] obtained a protein content included in the 11.0–40.9% range for *Conger myriaster* skin, by employing green extracting technologies.

### 2.2. Analysis of the FA Composition

A similar FA profile was detected in both eel tissues (Table 2). Thus, the two major FAs were C16:0 and C18:1ω9. Additionally, relatively abundant FAs were C18:0, C16:1ω7, C18:1ω7, C20:4ω6, C20:5ω3, C22:5ω3, and C22:6ω3. However, the comparative analysis of both tissues revealed remarkable quantitative differences. Thus, a higher content (*p* < 0.05) of C17:0, C20:1ω9, C22:1ω9, and C20:2ω6 was detected in the skin tissue. Contrary, C14:0, C16:1ω7, C20:4ω6, C22:4ω6, C20:5ω3, C22:5ω3, and C22:6ω3 revealed a higher presence (*p* < 0.05) in the muscle samples.

**Figure 1:**
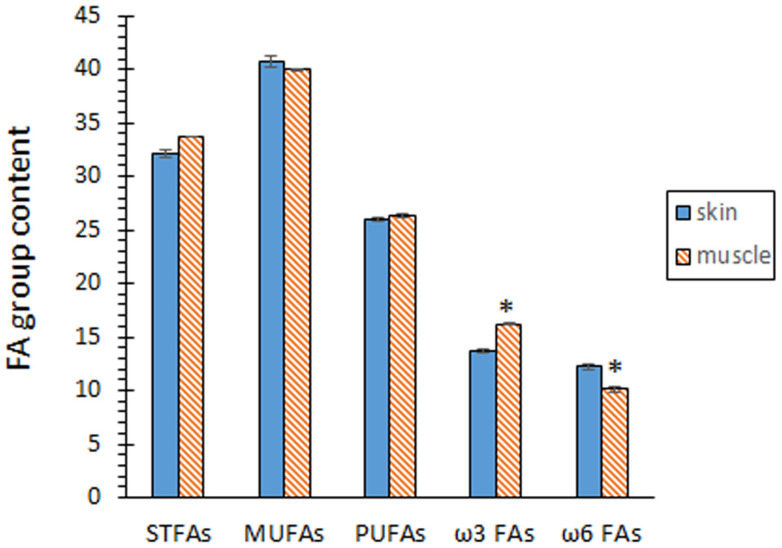
Fatty acid (FA) groups of eel skin and muscle (g·100 g^−1^ total FAs). Average values of four (*n* = 4) replicates; standard deviations are indicated by bars. Muscle values accompanied by an asterisk denote significant differences (*p* < 0.05) with skin values, according to the LSD test. Abbreviations: STFAs (saturated FAs), MUFAs (monounsaturated FAs), and PUFAs (polyunsaturated FAs).

**Figure 2:**
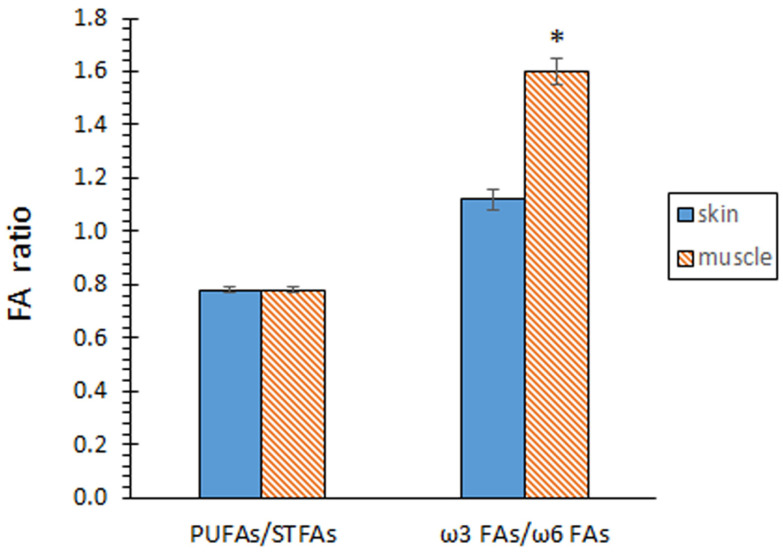
Fatty acid (FA) ratios of eel skin and muscle. Average values of four (*n* = 4) replicates; standard deviations are indicated by bars. Muscle values accompanied by an asterisk denote significant differences (*p* < 0.05) with skin values, according to the LSD test. Abbreviations as expressed in Figure 1.

Comparison of both kinds of tissues did not show significant differences (*p* > 0.05) in the contents of STFA, MUFA, and PUFA groups (Figure 1). According to the individual FA profile, the MUFA group was shown to be the most abundant (*p* < 0.05) in both tissues, while the PUFA group depicted the lowest (*p* < 0.05) presence. In agreement with this similar composition for the FA groups, no differences (*p* > 0.05) between both tissues could be outlined for the PUFA/STFA ratio (Figure 2).

The contents of total ω3 FAs and total ω6 FAs revealed remarkable differences between both tissues (Figure 1). Thus, samples corresponding to the skin tissue showed a lower content (*p* < 0.05) of ω3 FAs, but a higher content (*p* < 0.05) of ω6 FAs. As a result, a higher ω3/ω6 ratio (*p* < 0.05) was proved in samples corresponding to the muscle tissue (Figure 2).

A great interest has been paid to the presence of ω3 PUFAs, according to their beneficial health effects [48,49]. Based on epidemiological and clinical studies, EPA consumption has been related to circulatory, inflammatory, and coronary diseases [50], while DHA has been associated with the prevention of neurodegenerative diseases, foetal development, and the correct functioning of the nervous system and visual organs in the foetus [51]. Meanwhile, a relevant interest has also been given to the ω3/ω6 FA ratio [52,53]. Remarkably, recent studies have proved that Western populations do not include appropriate levels of ω3 FAs in their diet through natural dietary sources. In an attempt to avoid cardiovascular, neurological, and inflammatory concerns, the World Health Organization (WHO) recommends a higher ratio than 1:10 in the human diet [54]. Current results on both tissues have shown lower levels for EPA, DHA, and ω3 FAs than those present in the muscle of marine fish and invertebrate species [36,37,38]. However, results can be considered notably higher than in non-aquatic food such as poultry and egg [55], milk [56], and meat [57]. Remarkably, the ω3/ω6 ratio in both eel tissues was shown to be higher than 1 and was notably higher than 1/10, as recommended by the WHO [54].

No previous research is available regarding the FA composition of European eel (*A. anguilla*) skin. However, previous studies have focused on the FA composition of European eel muscle. According to the present results, a decreasing sequence for FA groups in muscle samples was described, i.e., MUFAs > STFAs > PUFAs, in individuals obtained in the northeastern Mediterranean Sea [26], in both wild and cultivated fish from Tunisian Mediterranean coasts [27], and in freshwater individuals from the River Ulla (Galicia, NW Spain) [58]. In such studies, and also in agreement with the current results, C18:1ω9 and C16:0 were the most abundant FAs.

Where FA ratios are concerned, lower PUFA/STFA ratios than in the present case were obtained in European eel (*A. anguilla*) muscle from the Mediterranean Sea (0.37) [26] and from both wild (0.46) and cultivated (0.52) individuals caught in the Tunisian Mediterranean coasts [58]. Regarding the ω3/ω6 ratio, higher values were detected in the muscle of freshwater individuals from the Ulla River (1.66–2.07) [27]. Additionally, Achouri et al. [58] found higher (3.28) and lower (1.31) ω3/ω6 ratio values in the muscle of both cultivated and wild individuals, respectively, obtained from Tunisian Mediterranean coasts.

Previous studies account for the FA composition of the skin and muscle tissues of related eel species. Thus, the same FA group distribution (MUFAs > STFAs > PUFAs) as in the current study was detected in the skin from *C. myriaster* eel from South Korea [47], in freshwater eel *A. japonica* muscle [41], in wild and cultivated Japanese eel (*A. japonica*) muscle [39], and in Japanese freshwater eel (*A. japonica*) muscle [42]. Contrary, the FA analysis of marbled eel (*Anguilla marmorata*) skin showed that C18:0 (ca. 50%) and C16:0 (ca. 22%) were the most abundant, with values for EPA and DHA being under 0.05% [59]; for the muscle tissue, this study proved that C18:1ω9 (ca. 45%) and C16:0 (ca. 19%) were the most abundant FAs, and EPA and DHA were present at a value below 0.03%. Regarding FA ratios, a higher ω3/ω6 ratio (4.48–5.41) than in the present work was detected by Park et al. [47] in *C. myriaster* eel skin from South Korea. Contrary, Lee et al. [41] obtained a similar ω3/ω6 ratio (0.90–1.67) in *A. japonica* muscle than in the current study.

Previous studies have also been focused on the FA composition of skin obtained from other fish species. During the analysis of the FA composition of skin obtained from three species from the Senegalese coast, Njinkoué et al. [43] proved that C16:0 and EPA were the most abundant (20.5% for both FAs) in *S. maderensis*, C16:0, C18:1ω9, and EPA (20.5, 15.5, and 10.4%, respectively) in *S. aurita*, and C16:0 and C18:1ω9 (28.4 and 12.5%, respectively) in *C. taeniops*; regarding DHA, values of 4.2, 2.5, and 6.9% were detected in such species, respectively. C18:1ω9 was the most abundant (ca. 36%), followed by C18:2ω6 (ca. 19%) and C16:0 (ca. 14%) in gilthead seabream (*S. aurata*) skin [44]; the presence of ω3 FAs was low (ca. 4 and 2% for DHA and EPA, respectively), so that a lower ω3/ω6 ratio (0.64) than in the present case was detected. The following FA distribution was detected by Ahmmed et al. [45] in blue mackerel (*S. australasicus*) skin: 19.5% (DHA), 17.3% (C16:0), 14.7% (C18:1ω9), and 2.30% (EPA); as a result, higher values than in the present study were obtained for PUFA/STFA (1.23) and ω3/ω6 (11.11) ratios. A very different FA distribution was found by the same authors [46] in king salmon (*O. tshawytscha*) skin; thus, C18:1ω9 was shown to be the most abundant (ca. 40%), followed by C16:0 (ca. 16%) and very low EPA and DHA values (<3%).

### 2.3. Determination of Lipid Classes

Results obtained for the composition of lipid classes is described in Table 3. Triacylglycerols (TAGs) were shown to be the most abundant lipid class in both tissues; values were included in the 400–412 g·kg^−1^ lipid range. A higher average content was observed in the muscle tissue; however, differences were not found significant (*p* > 0.05).

Free FAs (FFAs), compounds resulting from the hydrolysis of higher molecular weight compounds (i.e., TAGs and PLs) [60,61,62], provided values included in the 30–44 g·kg^−1^ lipids range; notably, higher values (*p* < 0.05) were detected in the skin tissue than in the counterpart edible substrate.

A remarkable presence of structured lipid classes (PLs and sterols, STs) were detected in the skin tissue (ca. 111 and 105 g·kg^−1^ lipids, respectively). Values were higher (*p* < 0.05) than those obtained in counterparts corresponding to the muscle tissue, especially for the ST compounds.

The analysis of the tocopherol composition of the current substrates indicated that the only tocopherol compound present was α-tocopherol. The content of this compound was found to be notably higher (*p* < 0.05) in the skin (274 mg·kg^−1^ lipids) than in the muscle (178 mg·kg^−1^ lipids).

Table 3 also indicates the lipid class content, expressed on a tissue basis. As for the previously mentioned results, on a lipid basis, higher (*p* < 0.05) FFA, PL, ST, and α-tocopherol values were detected in the skin tissue than in the counterpart muscle substrate. Notably, a higher average value of TAGs was observed in skin samples, although differences with muscle tissues were not significant (*p* > 0.05).

Based on their amphiphilic character, remarkable attention has been accorded to the PL compounds present in fish [5,6]. Thus, remarkable functions of PL compounds have been related to food production and pharmaceutical industries [63,64], these include antioxidant properties during food processing [65,66]. Based on their important role as lipid-soluble, chain-breaking antioxidants, tocopherol compounds have received great attention from marine technologists for their important role as lipophilic antioxidants [7,8]. Among them, α-tocopherol has been shown to be the most abundant in fish species [36]. The PL and α-tocopherol contents observed in the current eel by-product can be considered remarkable and correspond to the edible muscle of a medium-fat fish substrate [36,67], but lower than in a lean fish species [37,38].

Previous research regarding the analysis of lipid classes of European eel (*A. anguilla*) samples (skin or muscle) can be considered very scarce. According to the present results, TAGs were shown to be the most abundant lipid class in the muscle tissue from pre-migrant and migrant eel individuals [68]; in this study, phosphatidylcholine (PC) was shown to be the most abundant PL class. Regarding a related eel species, Park et al. [47] detected α-, (β + γ)-, and δ-tocopherol in *C. myriaster* skin from South Korea; as in the present case, α-tocopherol was shown to be the most abundant, with a content included in the 31–100 mg/100 g skin range. The presence of PL compounds was found to be relatively similar to the present concentrations in previous studies related to the skin substrate of other kinds of fish species. Thus, blue mackerel (*S. australasicus*) skin showed a 13.4 g·100 g^−1^ lipids concentration [45] and king salmon (*O. tshawytscha*) skin showed a 9.31 g·100 g^−1^ lipids value [46].

Previous studies also account for the lipid class analysis of the edible tissues of other eel species. Thus, Saito et al. [42] compared the lipid class composition of the initial and terminal stages of spawning migration of wild Japanese freshwater eel (*A. japonica*) muscle; as a result, TAGs were the major component in the initial-phase eels, but presented a remarkable content decrease in individuals corresponding to the terminal phase. A comparative study of the lipid class profile in both wild and cultivated individuals of the Japanese eel (*A. japonica*) was carried out by Oku et al. [39]; both in wild and cultivated individuals, TAGs were shown to be the most abundant lipid class of muscle (67.9–68.2%); other lipid classes detected were sterylesters (9.5–10.2%), FFAs (9.9–11.2%), STs (4.5%), PC (2.3–2.4%), and phosphatidylethanolamine (1.2–1.5%).

## 3. Materials and Methods

### 3.1. Solvents, Chemicals, and Standards

Solvents and chemical reagents used were of reagent grade and purchased from Merck (Darmstadt, Germany). The following solvents were employed: chloroform, methanol, hexane, toluene, ethyl ether, and isopropanol. In the case of tocopherol analysis, solvents used were liquid chromatographic grade. The following reagents were used: ammonium molybdate, hydroxylamine, cupric acetate, pyridine, acetic acid, acetic anhydride, acetyl chloride, and ferric trichloride.

Quantitative standards (1,2-dipalmitoyl-rac-glycero-3-phosphocholine, oleic acid, cholesterol, methyl stearate, nonadecanoic acid, and α-, β-, γ-, and δ-tocopherol) were purchased from Sigma-Aldrich (St. Louis, MO, USA). The following FAME standards were employed: Qualmix Fish (Larodan, Malmo, Sweden) and the Supelco 37 Component FAME Mix (Sigma-Aldrich, Laramie, WY, USA).

### 3.2. Fish Material and Sampling

European eels (*A. anguila*) (weight: 53–83 g; length: 32–39 cm) were captured during the authorised season (i.e., winter) and were slaughtered by ice water immersion at a local market (Mariscos Vivos del Grove, Quintela de Canedo, Ourense, Spain). Once at the post-slaughtered stage, eels were transported to the laboratory in insulated boxes on ice (0–1 °C). The fish (60 individuals) were distributed into four groups (fifteen individuals per group), which were considered independently for the statistical analysis (*n* = 4). As a first processing step, the fish were eviscerated and washed with running water. In each individual fish, the skin and muscle were excised and considered separately. Inside each of the four groups, portions corresponding to the same tissue (skin or muscle) were pooled together and subjected to the different chemical analyses.

### 3.3. Proximate Composition Analysis

Moisture content was determined as the weight difference in the homogenised tissue (1–2 g) before and after 4 h at 105 °C [69]. Results were calculated as g water·kg^−1^ tissue.

Crude protein content was measured using the Kjeldahl method [69] with a conversion factor of 6.25. Results were calculated as g protein·kg^−1^ wet tissue.

The crude lipid fraction was extracted using the Bligh and Dyer [70] method, which employs a single-phase solubilisation of the lipids using a chloroform–methanol (1:1) mixture. Results were calculated as g lipids·kg^−1^ wet tissue.

Ash content was measured according to the AOAC [69] method by heating the fish tissue at 550 °C. Results were calculated as g ash·kg^−1^ wet tissue.

### 3.4. FA Analysis

Lipid extracts were converted into fatty acid methyl esters (FAMEs) using acetyl chloride in methanol and then analysed using gas chromatography (Perkin-Elmer 8700 chromatograph, Madrid, Spain) according to an established procedure [71]. For it, a fused silica capillary column SP-2330 (0.25 mm i.d. × 30 m, Supelco, Inc., Bellefonte, PA, USA) was employed and the temperature program was as follows: increased from 145 to 190 °C at 1.0 °C min^−1^ and from 190 °C to 210 °C at 5.0 °C min^−1^; held for 13.5 min at 210 °C. The carrier gas was nitrogen at 10 psig and detection was performed with a flame ionisation detector at 250 °C. A programmed temperature vaporiser injector was employed in the split mode (150:1) and was heated from 45 to 275 °C at 15 °C min^−1^.

Peaks corresponding to FAMEs were identified by comparing their retention times with those of standard mixtures (Qualmix Fish and Supelco 37 Component FAME Mix). Peak areas were automatically integrated. C19:0 was used as an internal standard for quantitative purposes; for it, 100 μL (i.e., 40 μg C19:0) of a 0.4 mg·mL^−1^ solution in toluene were added to each sample before the methylation reaction with acetyl chloride. Limits of detection and quantification were 500 and 1500 area units, respectively. Quantitative calibration was carried out by means of the above-mentioned Supelco FAME Mix. Content of each FA was expressed as g·100 g^−1^ total FAs.

Results concerning FA groups (STFAs, MUFAs, and PUFAs; ω3 and ω6 FAs) and FA ratios (total ω3 FAs/total ω6 FAs and total PUFAs/total STFAs) were calculated taking into account the results obtained in individual FAs.

### 3.5. Analysis of Lipid Classes

To measure the TAG content, the total lipid extracts were first purified on 20 × 20 cm thin-layer chromatography plates coated with a 0.5 mm layer of silica gel G (Merck, Darmstadt, Germany) using a mixture of hexane-ethyl ether-acetic acid (90/10/1, *v*/*v*/*v*; two developments) as eluent [72]. Once the TAG fraction was purified, the method of Vioque and Holman [73] was used to measure the ester linkage content, according to the conversion of the esters into hydroxamic acids and their subsequent complexion with Fe (III). For quantification purposes, different quantities (0, 2, 5, 10, 20, and 40 μL) of a methyl stearate solution in toluene (41.0 mg/5 mL) were employed. The validity range was 16.4–328.0 μg methyl stearate and the R^2^ value of the analytical procedure was 0.9995. Results were calculated as g tristearine·kg^−1^ lipids and g tristearine·kg^−1^ tissue.

FFA content of the total lipid extracts was determined following the Lowry and Tinsley [74] method, which is based on the formation of a complex with cupric acetate-pyridine. In this study, benzene was replaced by toluene as organic solvent. For quantification purposes, different quantities (0, 5, 10, 20, 40, 60, 80, 100, 130, and 150 μL) of an oleic acid solution in toluene (705.3 mg/25 mL) were employed. The validity range was 0.5–15.0 μmol oleic acid and the R^2^ value of the analytical procedure was 0.9998. Results were calculated as g oleic acid·kg^−1^ lipids and g oleic acid·kg^−1^ tissue.

PLs were quantified by measuring the organic phosphorus in the total lipid extracts according to the Raheja et al. [75] method, which is based on a complex formation with ammonium molybdate. For quantification purposes, different quantities (0, 5, 10, 20, 40, 60, 80, 100, 130, and 150 μL) of a 1,2-dipalmitoyl-rac-glycero-3-phosphocholine (DPPC) solution in chloroform (15.3 mg/5 mL) were employed. The validity range was 16.1–483.0 μg DPPC and the R^2^ value of the analytical procedure was 0.9995. Results were calculated as g DPPC·kg^−1^ lipids and g DPPC·kg^−1^ tissue.

Free STs were determined on total lipid extracts using the method of Huang et al. [76] based on the reaction with acetic anhydride in acetic acid (Liebermann–Buchardt reaction). For quantification purposes, different quantities (0, 5, 10, 20, 40, 60, 80, 100, 130, and 150 μL) of a cholesterol solution in acetic acid (12.2 mg/5 mL) were employed. The validity range was 11.5–345.0 μg cholesterol and the R^2^ value of the analytical procedure was 0.9998. Results were calculated as g cholesterol·kg^−1^ lipids and g cholesterol·kg^−1^ tissue.

The content of tocopherol compounds was determined in both tissues according to the method of Cabrini et al. [77], with some modifications. For this purpose, a lipid fraction was carried out to dryness under nitrogen flux, dissolved in isopropanol, and analysed using HPLC (C18 5μm, 4.6 × 250 mm column; XBridge, Waters, Milfoird, MA, USA). The column was fluxed with methanol for 2 min; then, a gradient from 0 to 50% of isopropanol in 10 min was applied. A 1.5 mL·min^−1^ flow rate was employed and detection was carried out at 280 nm. The possible presence of α-, β-, γ-, and δ-tocopherol molecules was checked. For quantitative purposes, the content of each tocopherol compound present in the lipid extract was calculated with calibration curves prepared with the corresponding commercial tocopherol molecule (10–100 μL of a 1000 ppm tocopherol/methanol solution) and calculated as mg·kg^−1^ lipids and mg·kg^−1^ tissue.

### 3.6. Statistical Analysis

Data (*n* = 4) obtained from the different chemical analyses (proximate composition, individual FAs, FA groups and ratios, and lipid classes) were subjected to the ANOVA method to investigate differences between both tissues, i.e., skin and muscle (Statistica version 6.0, 200; Statsoft Inc., Chicago, Il, USA). A comparison of means was performed using the least-squares difference (LSD) test. The 95% confidence intervals of each chemical parameter were calculated; for it, the standard deviation of each sample and the number of replicates were considered.

## 4. Conclusions

The present study provides a first approach focused on the chemical composition of European eel (*A. anguilla*) skin. The presence of bioactive compounds in this waste substrate was comparatively analysed with the edible tissue, a seafood widely accepted by the consumer. Thus, higher (*p* < 0.05) levels of proteins (271.6 g·kg^−1^ wet tissue), lipids (38.0 g·kg^−1^ wet tissue), ash (27.7 g·kg^−1^ wet tissue), and ω6 FAs were observed in the skin tissues. Contrary, the muscle tissue showed higher (*p* < 0.05) moisture, ω3 FA, and ω3/ω6 values. Regarding lipid classes, higher proportions of PLs (111.1 g·kg^−1^ lipids), STs (104.7 g·kg^−1^ lipids), α-tocopherol (274.0 mg·kg^−1^ lipids), and FFAs (43.6 g·kg^−1^ lipids) were observed in the skin tissue. No differences (*p* > 0.05) between both tissues could be detected in TAG and FA group (STFAs, MUFAs, and PUFAs) values and in the total PUFA/total STFA ratio.

It is concluded that eel skin, a by-product resulting from commercial processing, can be considered a valuable source for the food and pharmaceutical industries by providing value-added constituents such as proteins, lipids, ω3 FAs, PLs, and α-tocopherol. Further research taking into account variations of the chemical composition resulting from internal and external factors ought to be addressed. The study agrees with the current search for alternative sources of healthy and nutritive compounds from waste substrates. As for the edible parts of fish, convenient handling and storage during the skin processing ought to be carried out to avoid the development of damage mechanisms such as autolysis, microbial activity, and lipid oxidation.

## Figures and Tables

**Table 1:** Proximate composition (g·kg^−1^ wet tissue) of eel skin and muscle ^1^.

Chemical Constituent	Tissue	
	Skin	Muscle
Water	677.0 ± 7.0	783.3 ± 3.5 *
Crude protein	271.6 ± 7.2	166.4 ± 1.8 *
Crude lipid	38.0 ± 0.9	28.6 ± 1.6 *
Ash	27.7 ± 2.1	9.9 ± 1.0 *

^1^ Average values ± standard deviations of four (*n* = 4) replicates. Muscle values followed by an asterisk denote significant differences (*p* < 0.05) with skin values, according to the LSD test.

**Table 2:** Fatty acid (FA) profile (g·100 g^−1^ total FAs) of eel skin and muscle ^1^.

FA	Tissue	
	Skin	Muscle
14:0	2.70 ± 0.03	3.09 ± 0.04 *
15:0	0.70 ± 0.06	0.65 ± 0.01
16:0	22.37 ± 0.15	22.39 ± 0.09
17:0	1.48 ± 0.05	1.05 ± 0.02 *
18:0	5.97 ± 0.13	5.91 ± 0.20
16:1 ω7	6.63 ± 0.12	6.92 ± 0.15 *
18:1 ω7	6.93 ± 0.06	6.84 ± 0.16
18:1 ω9	25.34 ± 0.40	25.09 ± 0.47
20:1 ω9	1.25 ± 002	1.12 ± 0.01 *
22:1 ω9	0.16 ± 0.01	0.13 ± 0.01 *
24:1 ω9	0.45 ± 0.03	0.15 ± 0.02 *
18:2 ω6	2.35 ± 0.05	2.25 ± 0.14
20:2 ω6	1.25 ± 0.05	1.05 ± 0.08 *
20:4 ω6	5.45 ± 0.09	4.25 ± 0.13 *
22:4 ω6	3.23 ± 0.14	2.44 ± 0.10 *
20:5 ω3	5.83 ± 0.14	6.74 ± 0.07 *
22:5 ω3	4.82 ± 0.18	5.14 ± 0.08 *
22:6 ω3	3.06 ± 0.08	4.23 ± 0.16 *

^1^ Average values ± standard deviations of four (*n* = 4) replicates. Muscle values followed by an asterisk denote significant differences (*p* < 0.05) with skin values, according to the LSD test.

**Table 3:** Composition of lipid classes of eel skin and muscle ^1^.

Lipid Class	Tissue	
	Skin	Muscle
Triacylglycerols	400.6 ± 28.0 (15.2 ± 3.0)	411.6 ± 7.3 (11.8 ± 0.6)
Free fatty acids	43.6 ± 1.4 (1.7 ± 0.4)	30.9 ± 0.8 * (0.9 ± 0.1 *)
Phospholipids	111.1 ± 5.5 (4.2 ± 0.6)	93.4 ± 5.5 * (2.7 ± 0.6 *)
Free sterols	104.7 ± 5.8 (4.0 ± 0.6)	24.2 ± 1.1 * (0.7 ± 0.2 *)
Alpha-tocopherol	274.0 ± 14.7 (10.4 ± 1.8)	178.0 ± 38.8 * (5.1 ± 1.9 *)

^1^ Average values ± standard deviations of four (*n* = 4) replicates. Data expressed as g·kg^−1^ lipids, except for alpha-tocopherol (mg·kg^−1^ lipids). Data in brackets indicate the content, on a tissue basis, expressed as g·kg^−1^ tissue, except for α-tocopherol (mg·kg^−1^ tissue). Muscle values followed by an asterisk denote significant differences (*p* < 0.05) with skin values, according to the LSD test.

## Data Availability

Data are contained within the article.
